# Moms in the NICU: developing a pilot to engage and empower women who have delivered a prematurely born infant

**DOI:** 10.1186/s12884-023-05738-8

**Published:** 2023-06-10

**Authors:** Kimber Padua, Rebecca Robinson, Amen Ness, Amy Judy, Grace M Lee, Jeffrey Gould

**Affiliations:** 1 Division of Neonatal and Developmental Medicine, Department of Pediatrics, Stanford University School of Medicine, Stanford, CA USA; 2 California Perinatal Quality Care Collaborative (CPQCC), Stanford, CA USA; 3 Division of Maternal Fetal Medicine, St. Elizabeth Medical Center, Boston, MA USA; 4 Division of Maternal-Fetal Medicine, Department of Obstetrics and Gynecology, Stanford University School of Medicine, Stanford, CA USA

**Keywords:** Mothers, NICU, Engage mothers, Empower mothers, Decrease risk of repeat preterm birth

## Abstract

**Background:**

Mothers spend long hours at their preterm infant’s bedside in the Neonatal Intensive Care Unit (NICU), giving clinicians the opportunity to engage mothers in caring for their own health.

**Objective:**

To develop a NICU based intervention to reduce the risk of a future premature birth by engaging and empowering mothers to improve their own health and identify barriers to implementing their improvement.

**Design:**

Development based on a framework of narrative discourse refined by the Quality Improvement Plan Do Study Act Approach.

**Setting:**

Level II Stepdown Neonatal Intensive Care Unit.

**Participants:**

14 mothers of preterm infants, ages 24–39 years.

**Methods:**

A team of Maternal Fetal Medicine Physicians, obstetricians, neonatologists, neonatal nurses, and parents developed guidelines to elicit the mother’s birth story, review the story with a clinical expert to fill in knowledge gaps, identify strategies to improve health to reduce the risk of future preterm birth, and facilitate mother developing an action plan with specific six week goals. A phone interview was designed to assess success and identify barriers to implementing their health plan. The protocol was modified as needed after each intervention to improve the interventions.

**Results:**

“Moms in the NICU” toolkit is effective to guide any clinical facilitator to engage, identify health improvement strategies, and co-develop an individualized health plan and its take home summary reached stability after the 5th mother. Mothers reported experiencing reassurance, understanding, and in some cases, relief. Participants were enthusiastic to inform future quality improvement activities by sharing the six week barriers faced implementing their health plan.

**Conclusion:**

Engaging in the NICU provides an opportunity to improve mothers’ understanding of potential factors that may be linked to preterm birth, and promote personally selected actions to improve their health and reduce the risk of a future preterm birth.

**Supplementary Information:**

The online version contains supplementary material available at 10.1186/s12884-023-05738-8.

## Introduction

Over the last several years, there has been a growing concern with the high rate of recurrent preterm birth [1–3]. Although a highly effective intervention is yet to be found, several strategies such as eliminating short interpregnancy intervals, providing aspirin to women at high risk for preeclampsia, and effective management of chronic hypertension and diabetes and appropriate use of progesterone and cerclage have been shown to reduce the risk of a repeat preterm birth [4–6]. Making women aware of these interventions and motivating them to overcome the barriers that may exist to obtain them is not an easy task. The importance of promoting optimal health during the postpartum and interconception periods has been recognized as an important strategy to improve the outcomes of subsequent pregnancies, reduce the risk of preterm birth, and improve long term women’s health [4, 6–9].

Although improving health before and between pregnancies is considered an important strategy, it has been difficult to achieve [7, 9]. For example, although postpartum visits offer the opportunity to treat ongoing or chronic conditions, promote optimal birth spacing, and promote optimal health behaviors, nationally less than 60% of women enrolled in Medicare or state children’s health insurance programs attend a scheduled postpartum medical visit [10, 11]. In a review of the literature, Jones et al. found that only 58% of women diagnosed with gestational diabetes attended follow-up within four months of delivery [12]. In California, under 50% of women insured by Medicaid attend their six week postpartum visit, and of the women that do attend their postpartum visit, only 47.5% receive contraception [13]. Although multiple barriers such as difficulties in obtaining transportation, financial issues, and scheduling have been described [10], the role of maternal motivation remains paramount.

Mothers often spend hours and days in the Neonatal Intensive Care Unit (NICU) at the bedside with their premature infants offering the opportunity to engage mothers to understand and act in ways which can reduce the risk of a subsequent preterm birth as well as ways to improve their own health. The purpose of the “Moms in the NICU” initiative was to develop and pilot an approach to motivating mothers to improve their overall health by providing mothers with a clear understanding of the circumstances of her preterm pregnancy, recommendations for future pregnancies, and recommendations for improving her own health, while caring for her preterm infant in the NICU. The first step challenge of a “Moms in the NICU” intervention was to develop a NICU based motivational interviewing approach [14, 15] utilizing the principles of empathetic listening and patient determined goal setting to both inform mothers of ways in which they could reduce the likelihood of a repeat preterm birth and improve their own health and to guide them in their selection of action steps, that were felt to be acceptable and potentially useful by both the clinical team and the participating mothers. This report will describe the development and initial piloting of the “Moms in the NICU” intervention.

## Methods

### Toolkit development

To support maternal health at the NICU bedside, we compiled content for a toolkit called “Moms in the NICU” through five focus group meetings with Maternal Fetal Medicine physicians (MFM), obstetricians, neonatologists, and neonatal nurses, three focus group meetings with the family advisory council for the NICU, and two interviews with NICU graduate parent advisors. When there was general agreement on the conduct, content, and scripting of the program, we began the development of our initial protocols. In developing the intervention, the NICU family advisory representatives played an important role in creating an overall approach and communication style designed to minimize feelings of guilt for having given birth to a premature infant. To better understand the significance of tailored interventions for mothers’ needs, a literature review on the perinatal use of motivational interviewing [15] as well as current interventions for prolonging the interpregnancy interval and the availability of alternative contraceptive medicine was conducted. Furthermore, existing health education materials were assessed for use and adapted for inclusion in this intervention. The intervention was designed to provide support and information such as the importance of appropriate birth spacing, management of ongoing chronic conditions, timely evaluation for prenatal care and providing important prior pregnancy information to the provider during the next pregnancy in order to reduce the likelihood of another preterm birth.

The intervention is based on two strategies: actively listening to mothers’ birth stories and co-creating a personalized health plan to take specific actions to improve her health. In our pilot, active listening was provided at an initial “health care facilitator” interview by a Clinical Research Coordinator and then at a medical expert interview conducted by a maternal fetal obstetrician. At this second interview the mother’s birth story was reviewed, questions were addressed, and a personalized health plan was co-developed. Prior to discharge, the mother and Clinical Research Coordinator reviewed the health plan, identified, and agreed upon specific six week goals. The Clinical Research Coordinator conducted a six week follow up phone interview to assess the mother’s satisfaction/dissatisfaction with the “intervention,” her success in meeting the six week goals, and the identification of any barriers she faced in meeting her goals. These barriers were entered into a database to inform future quality improvement initiatives.

Neonates requiring intensive care are first treated in Lucile Packard Children’s Hospital Level III NICU (neonatal intensive care unit). When their condition is stable, they are transferred to our level II step down unit. For our pilot, patients were recruited and the intervention took place in the step down unit, due to the likelihood that they were close to discharge. Following the completion of each intervention, we reviewed how well the protocol worked and made changes as needed based on the Plan-Do-Study-Act (PDSA) four step model for improving a process [16]. Following the revisions made after intervention number 5, the protocol was found to work well when the intervention was conducted with mothers 6 to 14. By number 14, we felt that the “Moms in the NICU” protocol was now suitable for formal evaluation and recruitment into the developmental pilot was stopped.

### Assessment of the intervention’s acceptance and perception of potential usefulness

A six week phone interview was conducted utilizing a set format in order to assess each participant’s assessment of the usefulness and acceptability of each aspect of the intervention (mothers’ story, meeting with the obstetric expert, designing personal health plan) and to assess the extent of their success and identify any specific barriers they encountered in implementing their six week health plan objectives. The recorded content of these interviews constituted the pilot’s qualitative outcome measures; content was reviewed by the project team following each interview to assess the acceptability of the intervention and to determine if further modifications to the protocol were needed.

A similar qualitative exit review addressing the effort required, the acceptability, and potential usefulness of the intervention was obtained from the two participating maternalfetal obstetricians.

### Study population

Mothers who spoke English and had a preterm infant who had been in the intensive care unit, did not experience any acute life-threatening complications, and was expected to be discharged from the step down NICU were asked to participate. The mother’s demographic and perinatal history was not considered prior to recruitment. Fourteen participants were recruited based on their preterm infant’s current post conception age (35–38 weeks corrected gestational age), and an anticipated discharge to home within 4–5 weeks. The mean gestational age of babies was 28.5 weeks. Out born infants were excluded due to the absence of maternal pregnancy and delivery information. The fourteen participants represented a diverse range of demographic and clinical profiles. They included racial and ethnic minorities, (Hispanic, Asian, and White, Black or African American and Other), both married and unmarried as well as U.S. and foreign-born women, whose education ranged from high school to graduate school.

Participants included first time mothers and second time mothers who had either singleton or multiple pregnancies. Delivery types included vaginal deliveries (spontaneous and induced) and Cesarean sections (elective and non-elective). The earliest delivery was reported at 25 gestational weeks and the latest delivery was reported at 32 gestational weeks. Table 1 presents indications for delivery and reported prenatal conditions among the 14 participants.

**Table 1:** Indications and Complications causing Early Delivery

Indications		Complications	
Premature Rupture of Membranes	64%	Pre-Eclampsia with Severe Features	28%
Artificial Rupture of Membranes	14%	Gestational Hypertension	7%
Spontaneous Rupture of Membranes	21%	Gestational Diabetes	35%
Preterm Labor	57%	Diabetes Mellitus Type 2	21%
Cervical Insufficiency	28%	Twin-Twin Transfusion Syndrome	7%
Maternal Hemorrhage	7%	Mono-Mono Pregnancy	7%
Bicornuate Uterus	7%	Limited Prenatal Care	7%

An important concern with respect to identifying mother’s health care actions that could reduce the likelihood of a repeat preterm birth was the possibility that the absence of these actions in the current pregnancy could make her feel guilty. To minimize and address this possibility, the interactions with mothers were carefully scripted to be supportive and minimize any feelings of guilt. Additionally, a preterm delivery is a traumatic event for many women [17]. Supportive empathetic and active listening were also deemed an essential component of the intervention to allow mothers to acknowledge their feelings that could include grief, anger, and guilt.

This project was reviewed and approved by the Institutional Review Board (IRB). The March of Dimes provided funding for the project.

## Results

### Toolkit development2

The final toolkit is presented in two volumes: (1) Project Planning and Implementation Strategies, and (2) Patient Tools. Through reflexive thematic analysis, our focus group participants agreed upon the following areas to be addressed: the identification of common risk factors associated with preterm births, the provision of support to mothers on understanding their own birth story, the highlighting of potential interventions that could reduce the risk of preterm birth in future pregnancies (such as avoiding a short interpregnancy interval, addressing hypertension or diabetes, etc.), and emphasizing the importance of early discussion with their obstetrician on key recommendations to follow during their next pregnancy.The handout and additional patient oriented educational materials were crafted with appropriate health literacy at the 8th grade reading level.

The toolkit and operational manuals addressed three essential stages of the intervention: [1] creation of a maternal birth story based on the details of the mother’s recent pregnancy, labor and delivery; [2] co-development of an individualized health plan with the mother to help support actionable items to improve her health and to reduce the risk of future preterm births including, but not limited to, guidelines on specific interventions and follow-up care; and [3] a six-week follow-up phone call to assess health plan implementation and identify barriers to its implementation. Table 2 shows the six key steps along with their rationale for the program. A complete set of operational manuals are available upon request.

**Table 2:** The “Moms in the NICU” six key steps and their rationale

**1. Enroll mother and initiate birth story.**	Allowing a mother to acknowledge her feelings about her experiences, which often can include grief, anger and guilt among parents of premature infants, may have a therapeutic benefit. Active listening begins to form a bond of concern and trust, as well as identifying areas that need to be discussed due to lack of information or misunderstandings.
**2. Abstract Maternal Pregnancy History.**	Provides key information to the Medical Expert which can then be compared to the mother’s birth story to identify areas not appreciated or misunderstood.
**3. Consultation with the Medical Expert.**	Continues active listening of birth story, answers questions about mother’s pregnancy, labor and delivery, filling in any gaps in understanding. Provides case specific health counseling recommendations, emphasizing targeted areas relevant to preterm birth prevention. Begins co-design of the health plan.
**4. Assemble Personalized Five Item Health Improvement Handout.**	This patient-centric packet of handouts includes: My Birth Story, Health Plan Recommendations, mother’s Plan for her health, optional Physician to Physician Letter and Medical Record Abstraction form.
**5. Co-design a Specific “Plan for My Health.”**	Identifies long term concerns and agrees upon specific activities that can realistically be accomplished within six weeks of her infant’s discharge. Provides health education materials to support the plan.
**6. Schedule Follow-up to Identify Successes and Barriers to Health Plan Activation.**	Evaluates a semi-structured phone interview six weeks after the meeting in the NICU to assess short term success of the intervention and record barriers as a basis for future community quality improvement initiatives.

#### Intervention staffing and their roles

Key roles for implementing the “Moms in the NICU” intervention included a health care facilitator and a medical expert.

### Health care facilitator (HCF)

In the pilot, this role was fulfilled by a Clinical Research Coordinator. The qualifications for this role include skills in active listening and general obstetric knowledge, and could be carried out by any health care professional meeting these qualifications. After the HCF identified and enrolled mothers in the program, a consultation with the Medical Expert was scheduled. During the recruitment and consenting process, the HCF asked preliminary questions about the birth story, beginning the therapeutic storytelling process. This prepared the mother in advance for the Medical Expert intervention. Additionally, this gave the HCF an initial report on what the mother understood during the labor and delivery, and what medical concepts or hospital protocols needed additional clarification. The same HCF was also responsible for the six-week follow-up phone call.

### Medical expert (ME)

In the pilot this role was filled by two MFMs, however, the role could also be filled by a general obstetrician, nurse practitioner, midwife or obstetric trainee with backup as needed. Mothers participated in a one-on-one consultation with the medical expert to share their birth story, clarify any concepts or issues related to their pregnancy and delivery, and discuss relevant interventions to improve their health and to reduce the risk of preterm birth during future pregnancies. The health care facilitator was present during the obstetric consultation to facilitate co-developing a health plan with the mother. Although optimal, this was not felt to be essential. While the medical expert was actively listening to the mother’s birth story, the health care facilitator took notes on issues, questions, and health conditions that the medical expert answered. This enabled the team to create a more complete birth story and input relevant information when assembling the handout and co-designing the health plan. The medical expert addressed the mother’s chronic conditions, discussed the importance of an appropriate interpregnancy interval, and made specific recommendations for the next pregnancy. The medical expert then made recommendations based on the discussion with the mother and a review of her medical record abstract. A personal health plan was then co-developed with the mother based on these recommendations.

### Qualitative intervention impressions

#### The birth story

When listening to the birth story, the two MFMs who participated in the pilot expressed surprise that despite the usual postpartum explanations of the delivery event many women still did not have a clear understanding of the circumstances of their pregnancy and delivery. This may be due to multiple factors that could interfere with mother’s ability to process information, including timing of the postpartum explanation they received and the notion that they may have been overwhelmed coping withtheir premature newborn. Mothers often found retelling their birth story multiple times to a healthcare professional to be an effective coping mechanism and expressed their gratitude for having an empathetic listener. After mothers told their birth story, the MFMs offered their compassion, context for medical interventions and clarification on hospital policies and protocols. Relief and appreciation from the mothers were observed after these thorough explanations. Although the birth story interventions took approximately an hour, the MFMs felt that updating the mother’s perception of her experience and her medical conditions was essential to providing effective perinatal care.

To assess the impact of the pilot’s birth story intervention, mothers were asked to recall and give feedback on this experience at the six week follow up interview. Universally, mothers reported experiencing reassurance, a better understanding, and in some cases, revelation and relief. They expressed a better understanding of why their baby may have been born prematurely and showed gratitude for the efforts of the MFM providers. Additionally, mothers reported appreciating a consultation with the MFM at a time and place (NICU bedside) that was convenient for them. See Table 3 for representative quotes.

**Table 3:** Selected Participant Feedback at the six-week follow-up interview

Domain	Quotation
**Better understanding of what occurred during Labor & Delivery**	*“It was helpful to go through my birth story with MFM. I wanted to make sure that I didn’t miss anything and everything I remembered was in fact what happened.”* *“It helped me to go through what was happening during the pregnancy and labor and delivery.”* *“I understand it wasn’t something I did wrong.”* *“When I was talking with Dr. XXX, she helped me realize how severe preeclampsia was and how the doctors were adjusting to my blood pressure. I understand that this was probably the cause of the preterm birth.”*
**Appreciation for additional Medical Expert (MFM) consultation**	*“Dr. XXX did a great job explaining everything and was really helpful in talking everything through with me.”* *“This process of telling my birth story and creating a health plan was helpful. I was able to ask questions and Dr. XXX answered all of them, and then I was able to read about the details which was very helpful.”*
**Understanding the importance of letting her body recover and the significance of an extended interpregnancy interval**	*“I will exercise more and have less stress. When I got pregnant, I was affected by the government shut down. I had two jobs – I started work at 4am and I wouldn’t finish until 10pm. So definitely having less stress will help. Dr. XXX told me to take baby aspirin and vitamins for future pregnancies.”* *“My husband is in Mexico, because we were affected by immigration issues. I haven’t started contraception yet, because I am not with my husband and we have not yet discussed future pregnancies. I was given pills when I was discharged from the hospital, but I haven’t started taking them yet. I thought I was interested in the IUD, but I wasn’t sure because my husband is away and if we wanted to have another kid, it just seemed like a lot of work to put in and take back out.”* *“This intervention made me think harder about the contraception piece. At my OB’s office, I was recommended progesterone-only pills, but I had never picked it up from the pharmacy. Hearing the importance of it made me reconsider and rethink it. Having been counseled on it again helped my decision to take the pills.”*
**Success with sharing their handouts and using the handout as a keepsake**	*“I gave the packet to my OB and he really liked it. He was asking me questions and when I didn’t know the answer, I pulled out my packet and read the details to him. He made a photocopy of the packet and put it in my file.”* An unexpected outcome of the handout was how one mother distributed it to her family as a means to share her story, because it was difficult, tedious and frustrating for her to retell her birth story: *“I was able to share my packet with my immediate family. It was a hard story to tell so it was helpful to have the packet to share. I didn’t want to share the story on social media, so it was nice to have this packet to tell family and friends.”* *“My brother, who lives in New York, bought me a calendar. It’s a baby’s first calendar and it has little spaces for birth story, baby’s weight, height and everything. I didn’t know answers to these off the top of my head, so I looked at my packet and all that information was in there. It is nice to have so that I can reflect on the birth story and remember what happened.”*

#### Co-designing the health plan

A personalized handout was created for each participant, which included both key elements of the birth story as well as all of the health improvement “recommendations” that had been discussed during the one-on-one consultation with the medical expert (Appendix 1). In co-designing a mother’s health plan, the health care facilitator first reviewed with the mother her understanding of the rationale and intended benefits of each recommendation. The health care facilitator then helped the mother to identify which current health problems were most relevant to her and assisted her in developing a personal health plan. An essential aspect of the health plan was that it contained an important objective (such as making an appointment for high blood pressure) that could be achieved by the six week follow-up. The initial handout approach was a listing of the health plan items on the handout. This was rejected by the first pilot mothers as being “too medical” resulting in their co-designing the format shown in Appendix 1.

At follow-up, mothers felt that co-designing their health plan helped improve their ability to care for their own health. This reaction suggested that the maternal engagement and empowerment we hoped to achieve during their one-on-one consultation was successful and had allowed mothers to recognize and specify actionable items on their Health Plan. Of the eleven mothers who were contacted at the six week follow up, eight had achieved their six week goals and two had attempted but encountered barriers to access specialized care.

An important goal of the pilot was to encourage a longer interpregnancy interval. However, not all mothers were receptive to our pilot’s approach to contraceptive counseling with respect to long-acting reversible contraception (LARC), claiming that they wanted something more natural or had insurance issues with obtaining LARC. In moving forward from the pilot, further work on how to best perform contraceptive counseling with respect to addressing cultural social differences and personal preferences, could strengthen this goal.

#### Qualitative findings from the six-week follow-up with mothers

Our initial objective of the six-week follow-up was to assess the extent to which the actionable items had been carried out. This was not met with enthusiasm, and we were unable to set up follow up interviews for each of the first three participants due to the mother’s conflicting priorities and lack of further interest in participating in the pilot. We then shifted the focus from what the mother had achieved to what were the barriers that she faced in trying to achieve her goals. We created a database to formally record the specific barriers faced that could serve as a starting point for local quality improvement initiatives (Appendix 2). We then presented the six-week follow-up as an opportunity to identify the barriers that had been experienced as a first step towards their removal. This partnership approach was very successful in large part because the mothers expressed having faced many barriers to receiving care in the past and were enthusiastic about providing their experiences to inform quality improvement. The identification of barriers and their inclusion became actionable items and an important goal of the six-week follow-up. The barriers included lack of transportation, difficulty setting up appointments, lack of childcare, and no specialized care available. It also revealed serious barriers that we had not anticipated: overwhelming infant needs, competition between mother’s appointments and baby’s appointments, and fatigue that made it difficult to “stay on the phone and put out all the effort required to make my appointments.”

An important role of the six week follow-up was to obtain a qualitative assessment of the mothers ’usefulness and acceptability of each aspect of the intervention. In summary, mothers reported high satisfaction with their participation. Representative excerpts of the participants’ responses are shown in Table 2. Although the number of participants in the pilot was too small to formally assess its effectiveness, of the eleven mothers who participated in the six week follow-up call, eleven kept their six week postpartum check-up, eight started on contraception, eight met their six week goals, and two attempted but faced barriers in obtaining specialty appointments. Some of the mothers remembered to bring their handouts with them to their six week postpartum check-ups and were given both positive and negative feedback from their primary obstetricians. One of the mothers was recommended to have biweekly cervical length ultrasounds during the next pregnancy starting at 16 weeks until 24 weeks by the medical expert on our team but her primary provider did not accept this approach. This information allowed us to revisit the approach of engaging the mother’s primary obstetrician. We reemphasized the collaborative nature of our recommendations to optimize the outcome of future pregnancies based on medically necessary and evidenced based postpartum and timely prepartum care, and not as a critique of their care. Through PDSA, the intervention added a step to include a physician-to-physician letter to address this, and further promote maternal health care through collaboration.

## Discussion

Over the last several years, there has been a growing concern with the high rate of recurrent preterm birth [1–3]. Although the postpartum appointment is considered an important strategy to identify and manage physical and mental health conditions, promote optimal birth spacing, and promote optimal health behaviors that would improve maternal health and decrease the likelihood of preterm birth [4, 6–8] the rates of postpartum appointment attendance have been disappointingly low.

Experiencing a preterm delivery is a traumatic event for many women [17]. An important aspect of the intervention was to educate the medical expert with respect to the importance and need for empathetic active listening and storytelling [18], to encourage mothers to acknowledge their feelings about their experience, which often included grief, anger and guilt. Studies indicate that empathetic listening not only provides comfort but also leads to better therapeutic results and has been used to promote postpartum care and interconception health [14, 15]. This open-ended approach provided an opportunity to decrease the negative impact of their preterm birth as well as to co-design a plan to improve their health. It also brought to light many unexpected knowledge gaps and misconceptions regarding their pregnancy and delivery. Although this intervention took approximately an hour of the medical experts’ time, it laid a foundation of encouragement and empowerment for action as reflected in the positive feedback that we obtained at the six-week follow-ups.

Initially we viewed the six-week follow-up as an opportunity to evaluate if our intervention had motivated the mother to complete her health plan. However, we quickly learned that success in carrying out her plan was not primarily dependent on mother’s motivation. The main determinants were the many barriers she faced. We therefore expanded the intervention by incorporating, as a quality improvement component, a database designed to document the extent of the specific barriers faced in trying to achieve her health plan. This shift in emphasis was received enthusiastically by the pilot mothers, who had experienced many health care barriers in the past, and now saw themselves as part of a quality Improvement team to document these barriers as a first step towards their “removal.”

### Limitations

The pilot study had several limitations. The first is that we only included English-speaking mothers whose infants did well and our handout and additional educational materials were at the 8th grade rather than the recommended 5–7 grade level [19]. We cannot be assured that the positive reactions that we got during this pilot would also hold true for non-English speakers, or mothers whose infants’ courses were extremely difficult. Furthermore, exploring the possibility of using the mothers in the NICU approach as means to address equity was not adequately utilized in this pilot. Finally, the pilot was developed in a single clinical setting and although participant feedback was very encouraging, its success in bringing about health promoting behavior change was not formally evaluated. Assessment in several additional sites is warranted to assess what adaptations to the approach, if any, will be required for implementation in these new settings, and to conduct a formal evaluation of its effectiveness in promoting health improvement. Although we developed a very specific and detailed approach, our pilot demonstrates that the model of using the time a mother spends with her infant in the NICU to promote the importance of postpartum care and to co-develop a plan for health improvement including reduction of future preterm births is worth pursuing. We also identified that even when motivated to complete their health plan our mothers faced many barriers. Moving forward we recognize that developing effective strategies to identify and overcome potential barriers to healthcare access is an essential goal in the design of any intervention to improve maternal health.

## Conclusion

In conclusion, the “Moms in the NICU” initiative is a new model to promote maternal engagement, empower mothers to improve their health, and reduce their risk of having a further preterm birth. Our pilot approach was well received by the participating mothers and health care professionals and deserves further beta testing.

## Electronic supplementary material

Below is the link to the electronic supplementary material.

Supplementary Material 1

## Data Availability

The datasets used and/or analyzed during this study are available from the corresponding author on reasonable request.
