# Supplementary material for: Moms in the NICU: developing a pilot to engage and empower women who have delivered a prematurely born infant
Source: BMC Pregnancy Childbirth. 2023 Jun 10;23:432. doi: 10.1186/s12884-023-05738-8 (PMC10257253; doi:10.1186/s12884-023-05738-8)
Supplement: Supplementary file 1 — Supplementary Material 1 [file 12884_2023_5738_MOESM1_ESM.docx]

**Appendix Legend**

**Appendix 1. My Birth Story Template**

**Appendix 2. Health Plan Recommendations Template**

**Appendix 3. Plan for My Health Template**

**Appendix 4. Physician to Physician Letter Template**

**Appendix 5. Medical Abstraction Form**

**Appendix 6. Example of Handout**

**Appendix 7. Co-designed Health Plan Template**

**Appendix 8. Database**

**Appendix 1. My Birth Story Template**

| **My Baby’s Birth Story**  A record of what happened during the birth of my baby. |
| --- |
| **Prior Health Conditions and Previous Pregnancies:**   - Where did you receive prenatal care? Who was your OB? - Is this your first pregnancy? If no, how many previous pregnancies? - Were your previous pregnancies carried to term? - Any complications in your previous pregnancies? - Multiple gestation? - Any previous surgeries? Specifically, any abdominal or vaginal surgeries?   **Health Conditions:**   - Hypertension - Type 2 DM - Autoimmune diseases   **During Pregnancy:** [Insert pertinent information about pregnancy management, delivery management, and potential factors associated with preterm birth]   - Normal Ultrasounds? - Any abdominal trauma? - Any infections? - Multiple marker test? NIPT? Amniocentesis? - Gestational Diabetes? - Preeclampsia? - Preterm Labor or Rupture of Membranes?   **Labor and Delivery:** [Record the mother’s version of what happened during labor and delivery.]   - Spontaneous vs. Medically indicated - Vaginal delivery vs. C-section   **My Baby’s Information:** [Insert pertinent information about baby, e.g. DOB, Apgar score, weight, gestational age.]  Name:  Date of Birth:  Gender:  Gestational Age at Birth:  Birthweight: (kg and lbs/oz)  Length: (cm and inch) Head Circumference: (cm and inch) |

| **Letting My Body Recover**  What my body needs to recover from my recent pregnancy and labor. |
| --- |
| [Insert standard information about IPI and LARC, importance of taking time to heal between pregnancies.]  The length of time your body has to recover between pregnancies is important for all women. The longer your interpregnancy interval, the better chance you have to optimize your body’s nutritional status and ensure healing by allowing your muscles to recover. Mothers who delivery prematurely and have another birth with 18 months have a much higher risk of a repeat preterm birth.  *Recommendation:* Family planning using highly effective contraceptives for the next year is strongly recommended. These methods include IUDs and implants. Birth Control pills can also be considered.  *My Plan****:*** *[Create a Health Plan for Mom for IPI and LARC. Use free text to personalize information]*  Review contraceptive options (see fact sheet on birth control choices).  Make an appointment with my doctor to discuss options for effective contraception.  Wait at least 18-24 months before trying to get pregnant again.   - _______________________________________________________________________ - _______________________________________________________________________ - _______________________________________________________________________ - _______________________________________________________________________ |

**Appendix 2. Health Plan Recommendations Template**

| **Taking Care Of My Health Now**  What do I need to do to take care of my health right now? |
| --- |
| [Insert information on how to manage any chronic conditions that the mother has (diabetes, hypertension, etc.) or reduce harmful behaviors (smoking cessation, etc.). Generic text inserted below – please delete what is not relevant;  **Diabetes**. Gestational diabetes is high blood sugar (glucose) that is first diagnosed during pregnancy. Having gestational diabetes in your last pregnancy increases your risk of having gestational diabetes in another pregnancy and of having type 2 diabetes later in life. While you may not have any symptoms of high blood sugar, diabetes can lead to many health problems, such as high blood pressure, heart disease and kidney disease.  Recommendations: Because you had gestational diabetes while you were pregnant, you should be retested for diabetes after your baby is born. Please have the test approximately 6 weeks after your baby’s birth date or _________, and every 1-3 years after giving birth. Breastfeeding is helpful for reducing your risk of developing type 2 diabetes after pregnancy. Eating healthy foods and being active every day can also help to get to and stay at a healthy weight to help reduce your risk of getting type 2 diabetes.  My Plan:   - Call my doctor’s office to schedule a screening test (fasting plasma glucose or oral glucose tolerance test) for diabetes about 6 weeks after my baby’s birth. Remember not to eat anything after midnight of the night before my scheduled test. - Remember to get screened every 1-3 years. More frequent screening is needed if I have prediabetes.   **Hypertension**.  High blood pressure that first occurs in the second half (after 20 weeks) of pregnancy is called ***gestational hypertension or preeclampsia***. Management depends on how high your blood pressure is. Most women have only a mild increase in blood pressure. Some women, however, develop severe hypertension and are at risk of serious complications. All women with gestational hypertension are monitored closely to make sure their blood pressure does not go too high and to look for signs of preeclampsia. If you have preeclampsia you are usually hospitalized.  You are at greater risk of high blood pressure if you:   - have family history of hypertension - are overweight - are not physically active - smoke cigarettes - drink more than two alcoholic drinks per day - eat a poor diet (too much fat, not enough fruits and vegetables) - eat too much salt   Recommendations: Lifestyle habits can affect blood pressure. If lifestyle changes alone do not lower your blood pressure, medications usually are recommended. Many types of medications are available that work in different ways. It is important to continue taking your medication even when you are feeling healthy. It also is important to continue your healthy lifestyle habits even if taking medication lowers your blood pressure readings into the healthy range. When you are not pregnant, high blood pressure may have no symptoms and can, over time increase your risk of heart attack, stroke, and kidney disease.  My Plan: Adopting certain lifestyle habits can decrease your risk of developing or controlling high blood pressure:     - Try to lose weight if you are overweight. - Limit your intake of alcohol. - Exercise regularly. At least 3 times a week. - Cut back on salt. - Change your diet—The DASH (Dietary Approaches to Stop Hypertension) eating plan focuses on heart-healthy foods which has been shown to lower blood pressure. - Check your blood pressure regularly and follow up with your doctor to see if you need medication. The goal is a normal blood pressure is below 130/80. - Before your next pregnancy, speak with your OB and make sure your condition is under good control and you are on medications that are safe in pregnancy.     ***Smoking***. Babies who are exposed to secondhand smoke, from you or others around you who smoke, are more likely to get sick than babies who are not exposed. Also, babies exposed to smoke have a greater risk of dying from sudden infant death syndrome (SIDS). Even third hand smoke, or smoke that is left in carpets and furniture where a person once smoked, can be harmful to your baby’s health.  Recommendations: Smoking is the one of the most important modifiable causes of poor pregnancy outcomes in the United States, and is associated with maternal, fetal, and infant morbidity and mortality.  My Plan:   - Stop smoking if you are a current smoker. - If you haven’t quit, don’t smoke around your baby. - Don’t allow your partner or other people to smoke around your baby. - Choose activities where your baby isn’t exposed to secondhand smoke. - Leave the room with your baby if others are smoking. - Spend time with non-smokers.   **Postpartum Recovery Period:**  You may be experiencing some symptoms such as backaches, sore nipples, and vaginal pain. Most of these symptoms will be temporary and will gradually ease up within the first six weeks of recovery. While you’re focusing your care and attention on your newborn, it is important to remember to allow your body to recuperate from the monumental task of carrying a pregnancy.  **Breastfeeding:**  Breastfeeding can benefit both moms and babies. Moms can reduce their chances of breast cancer, ovarian cancer, diabetes, and cardiovascular disease. Breastfeeding can also help women lose weight gained during pregnancy.  Recommendations:   - Stay relaxed. This will keep oxytocin pumping in your body and allow you produce more breast milk. Remember to be patient while you wait for your milk to come in. - Continue taking prenatal vitamins or folic acid supplementation. - Drink a lot of fluids to stay hydrated. - Work with a lactation consultation for guidance.     **Sore Nipples from Breastfeeding:**  If you experience tenderness, soreness and cracking in your nipples, it is normal after pregnancy. Remember to position your baby properly (facing the breast), and make sure the baby has latched onto your areola.  Recommendations:   - Use warm water on your nipples in the shower, but remember soap, fragrance and alcohol can irritate your skin. Use warm compress (washcloth) 10 minutes before breastfeeding. - Try different positions: cradle hold, cross cradle hold, football hold. - Apply a few drops of breast milk on the affected nipple before breastfeeding. After breastfeeding, apply some milk again. - Work with a lactation consultation for guidance.   **Exercise:**  Usually, it is safe to begin exercising a few days after giving birth—or as soon as you feel ready. If you had a ***cesarean delivery*** or other complications, ask your health care provider when it is safe to begin exercising again.  Exercise has the following benefits for postpartum women:   - It helps strengthen and tone abdominal muscles. - It boosts energy. - It may be useful in preventing postpartum depression. - It promotes better sleep. - It relieves stress.   Recommendations:   - When you are ready to start exercising, walking is a great way to get back in shape. Walking outside has an added bonus because you can push your baby in a stroller. There are special strollers made for this kind of activity, but using a regular stroller is fine. - Another good way to get daily exercise is by joining an exercise class. Working out with a group and socializing with group members can help keep you motivated. - Aim to stay active for 20–30 minutes a day. Remember, even 10 minutes of exercise benefits your body. When you first start exercising after childbirth, try simple postpartum exercises that help strengthen major muscle groups, including abdominal and back muscles. - Gradually add moderate-intensity exercise. If you exercised vigorously before pregnancy or you are a competitive athlete, you can work up to vigorous-intensity activity. Stop exercising if you feel pain.   **Healthy diet:**  Maintaining a healthy diet can help promote healing and recovery. Always try to eat a variety of healthy foods.  Recommendations:   - Eat a healthy and balanced diet with protein, fruits and vegetables. - Avoid sugary foods, spices, citrus fruits, caffeine, smoked meats, and alcohol. - Drink a lot of fluids to stay hydrated. - Foods that will increase your breastmilk: asparagus, apricots, basil, dill leaves, spinach, carrots, salmon, almonds, oatmeal, barley, chickpea, brown rice     **Vaginal Soreness and Bleeding:**  It is normal to experience some vaginal soreness and irregular vaginal bleeding for the first six weeks after giving birth. Your flow will be irregular or absent, which is normal as well, especially if you are breastfeeding. After six weeks, if you have a heavy flow (ie. Soaking a pad once an hour) along with any fever, chills, or bad smelling vaginal discharge, please call you OB.  Recommendations:   - Use sanitary napkins as needed. - Sit on a pillow or padded ring. - Pour warm water on your perineum. - Sit in a warm bath just deep enough to cover your buttochs and hips for five minutes. - If you have a wound (from an extensive tear), you can cool it down using an ice pack.   **Abdominal Cramps:**  It can take up to six weeks for your uterus to shrink back to its normal size. Some women experience afterpains, which often resemble menstrual cramps. If your pain does not improve after the 6 – 8 weeks, please call your OB.  Recommendations:   - You can take acetaminophen (Tylenol) every 6 hours for cramping. - Walking around reduces bloating and pressure in your intestines and abdomen.   **Pain around C-section incision:**  Pain from your c-section can last up to 8 weeks.  Recommendations:   - You can take acetaminophen (Tylenol) every 6 hours for cramping. - Avoid heavy lifting! - Check your incision site. If you notice any drainage, call you OB.   **Difficulty Urinating:**  Sometimes women have difficulty urinating after giving birth, which is normal. The ordeal your body has just gone through, pregnancy, labor and delivery, can stretch or even injure your pelvic floor muscles.  Recommendations:   - Pour some warm water over your perineal area helps to stimulate voiding. - Taking a sitz bath can also help with difficulty with urination, as it promotes blood flow and healing to the perineal and vaginal area. - Do Kegel exercises to help strengthen your pelvic floor. - If you would like to induce urgency, apply an ice pack to your perineal area. - If you still can’t urinate call your doctor or a consult a Urogynecology specialist.   **Constipation:**  You may experience some pain or discomfort accompanied by lumpy stools and a sense of incomplete bowel evacuation. If you are taking narcotics (such as Vicodin or Percocet) for postpartum pain, they may be the reason why you are feeling constipated, because they slow down your digestive system.  Recommendations:   - DON’T STRAIN. - Remember to drink plenty of fluids. - Eat fiber-rich foods. - Go for walks. - Stool softeners and mild laxatives are available at your local pharmacy.   **Hemorrhoids:**  After your deliver – especially after a vaginal delivery, many women develop hemorrhoids. Approximately 25% of women who develop hemorrhoids after having a baby still have them up for up to six months.  Recommendations:   - Sitz baths – soak your anal area in plain warm water for 10 – 15 minutes, 2 – 3 times per day - Topical anesthetics - Witch hazel pads - Glycerin and hydrocortisone suppositories.   **Fatigue:**  It is inevitable that you will feel physical and mental fatigue after having a baby. Although it is easier said than done, practice good sleep hygiene.  Recommendations:   - Ask family members for help so that you can focus on feeding the baby and going back to sleep. - If you have to care for other children, it is OK to ask friends and family for help. - Try to take a nap when your baby naps. - When it’s time to breastfeed, sit with your feet up and get as comfortable as you can. Or breastfeed in the side-lying or laid-back nursing position. - Let the housework go for a while. You can catch up when you’re feeling up to it. Have your partner help with household chores. - Limit guests and visitors. - Eat a health well-balanced diet. - Stay Hydrated. Avoid drinking too much caffeine. - If you are struggling and continue to have no energy even with good nutrition and rest, please contact your doctor. Ask your doctor about postpartum depression, anemia, and hypothyroidism.   **Postpartum Blues vs. Postpartum Depression:**  Postpartum Depression affects up to one in seven women. It is important for you to take care of your emotional health. Focus on resting and bonding with your baby.  Postpartum Blues: About 2–3 days after childbirth, some women begin to feel depressed, anxious, and upset. They may feel angry with the new baby, their partners, or their other children. They also may cry for no clear reason, have trouble sleeping, eating, and making choices, and question whether they can handle caring for a baby. These feelings, often called the ***postpartum blues***, may come and go in the first few days after childbirth. The postpartum blues usually get better within a few days or 1–2 weeks without any treatment.  Postpartum Depression: Women with ***postpartum depression***have intense feelings of sadness, anxiety, or despair that prevent them from being able to do their daily tasks. Postpartum depression can occur up to 1 year after having a baby, but it most commonly starts about 1–3 weeks after childbirth.  Postpartum depression probably is caused by a combination of factors. These factors include the following:   - Changes in ***hormone***levels: Levels of ***estrogen***and ***progesterone***decrease sharply in the hours after childbirth. These changes may trigger depression in the same way that smaller changes in hormone levels trigger mood swings and tension before menstrual periods. - History of depression: Women who have had depression at any time—before, during, or after pregnancy—or who currently are being treated for depression have an increased risk of developing postpartum depression. - Emotional factors: Feelings of doubt about pregnancy are common. If the pregnancy is not planned or is not wanted, this can affect the way a woman feels about her pregnancy and her unborn baby. Even when a pregnancy is planned, it can take a long time to adjust to the idea of having a new baby. Parents of babies who are sick or who need to stay in the hospital may feel sad, angry, or guilty. These emotions can affect a woman’s self-esteem and how she deals with stress. - Fatigue: Many women feel very tired after giving birth. It can take weeks for a woman to regain her normal strength and energy. For women who have had their babies by C-section, it may take even longer. - Lifestyle factors: Lack of support from others and stressful life events, such as a recent death of a loved one, a family illness, or moving to a new city, can greatly increase the risk of postpartum depression.   Recommendations:   - If you feel this way it is important to **contact your OB or mental health professional**. - Talk with a mental health professional; often talk therapy in combination with medication is helpful for moms. - Sometimes, one-on-one therapy is needed for only a few weeks, but it may be needed for a few months or longer. - Group therapy is a good opportunity to meet with a therapist and other people with problems similar to yours. - Family therapy for you and your family members - Couples therapy for you and your partner - Support groups can be found at local hospitals, family planning clinics, or community centers. - The hospital where you gave birth or your health care provider may be able to assist you in finding a support group.   Useful information about postpartum depression can be found on the following web sites:  National Women’s Health Information Center <http://www.womenshealth.gov/mental-health/illnesses/postpartum-depression.html>  Postpartum Support International [www.postpartumsupport.net](http://www.postpartumsupport.net/)  Medline Plus <http://www.nlm.nih.gov/medlineplus/postpartumdepression.html>  *My Plan****:*** *[Create a Health Plan for Mom for health conditions she can address right now. Use free text to personalize information]*   - _______________________________________________________________________ - _______________________________________________________________________ - _______________________________________________________________________ - _______________________________________________________________________ |

**Appendix 3. Plan for My Health Template**

| **Planning For Future Pregnancies**  What do I need to know if I get pregnant again? |
| --- |
| [Insert reminder to seek early prenatal care and any information about her previous pregnancy and health status that she should tell her OB/GYN.]  **Preconception and Prenatal Care**:  Having a healthy pregnancy is one of the best ways to promote a healthy birth. Your health in the early weeks of your pregnancy is particularly important for your baby’s health. Take good care of yourself. Anything you do that harms your body can also harm your baby. Health care providers may also do blood tests and imaging tests early on, particularly for women who previously had a preterm birth.  **Vitamins:**   - Before getting pregnant it is important to take at least 400mcg of folic acid daily. This can be in a prenatal vitamin or alone if prenatal vitamins make you nauseated. - Folic acid supplementation helps prevent spinal cord defects (like Spina Bifida) when taken before and in early pregnancy.   **Tips for a Healthy Diet in Early Pregnancy:**   - Eat a balanced diet. Make sure your diet includes plenty of beans, peas, and leafy green vegetables. - Do not skip meals or go for many hours without eating. If you are nauseated, try to eat a small, healthy snack every 2 to 3 hours. - Do not eat fish that has a high level of mercury, such as shark, swordfish, or mackerel. Do not eat more than one can of tuna each week. - Drink plenty of fluids, enough so that your urine is light yellow or clear like water. If you have kidney, heart, or liver disease and have to limit fluids, talk with your doctor before you increase the amount of fluids you drink. - Cut down on caffeine, such as coffee, tea, and cola. - Do not drink alcohol, such as beer, wine, or hard liquor. - Fortified cereal and whole wheat bread are good additional sources of folic acid. - Increase the calcium in your diet. You may also take calcium supplements and choose foods such as cheese and yogurt.   **Lifestyle:**   - Get plenty of rest. - Get at least 30 minutes of exercise on most days of the week. Walking is a good choice. If you have not exercised in the past, start out slowly. Take several short walks each day. - Do not smoke. If you need help quitting, talk to your doctor about stop-smoking programs. These can increase your chances of quitting for good. - Do not touch cat feces or litter boxes. - Wash your hands after you handle raw meat, and fully cook all meat before you eat it. - Wear gloves when you work in the yard or garden, and wash your hands well when you are done. - Cat feces, raw or undercooked meat, and contaminated dirt can cause an infection that may harm your baby or lead to a miscarriage. - Do not use saunas or hot tubs. Raising your body temperature may harm your baby. - Avoid chemical fumes, paint fumes, or poisons. - Do not use illegal drugs or alcohol.   Recommendations: Caring for your own health before you become pregnant and early pregnancy is good for you and for your baby. Pre-conception and prenatal care can help prevent complications and support a healthy pregnancy.   - Maintain a healthy weight. Obesity (BMI >30) and being underweight (BMI<19) are both associated with preterm birth. - If you smoke or use recreational drugs, stop. - Avoid alcohol. There is no known safe amount of alcohol in pregnancy.   *[Insert any of the following diagnoses from the birth story that are relevant for the Mom and her health status that she should tell her OB/GYN]*  **Cervical Insufficiency**. In some women, the cervix can shorten and open too early in pregnancy with little or no labor pains. Early additional screening for signs of cervical insufficiency during the early part of your next pregnancy may help to identify potential interventions to reduce recurrent preterm birth. Large studies have shown that weekly injections of 17-hydoxy progesterone starting at 16 weeks gestation may help in reducing the chances of repeat preterm birth by as much as one-third.  If you have had this problem in your prior pregnancy it is recommended you see a Maternal Fetal Medicine (High Risk Pregnancy specialist) before or early in you next pregnancy.  Recommendation**:**   - Option 1: Starting at 16 weeks - Transvaginal ultrasound cervical length weekly or biweekly until 24 weeks - If cervical length <25 mm, a cervical cerclage (a banding stitch around the upper cervix) can be placed to help keep the opening to your womb closed throughout your pregnancy. - Option 2: At 13-14 weeks - Cerclage (a banding stitch around the upper cervix) can be placed to help keep the opening to your womb closed throughout your pregnancy. - With either option begin 17 alpha hydroxy progesterone 250 mg IM injections weekly beginning at 16 weeks till 36 weeks.   **Spontaneous Preterm Labor and Preterm Rupture of Membranes**. Although spontaneous preterm (early) labor is by far the most common cause of preterm birth, we do not have a good understanding why some women have early labor or early rupture of membranes. But once it happens, we do know that women are at higher risk (15-25%) for having another early delivery in the future. Large studies have shown that weekly injections of 17-hydoxy progesterone starting at 16 weeks gestation may help in reducing the chances of repeat preterm birth by as much as one-third.  Recommendation**:**   - Starting at 16 weeks, begin 17 OH progesterone 250mg IM weekly until 36 weeks. - Transvaginal ultrasound cervical length weekly or biweekly until 24 weeks - If cervical length <25 mm, a cervical cerclage (a banding stitch around the upper cervix) can be placed to help keep the opening to your womb closed throughout your pregnancy.   **Severe Hypertension.** Early-onset pregnancy-related hypertension and preeclampsia can increase chances of a preterm birth and may also affect the growth of the fetus. Though we may not understand why certain women develop hypertension during pregnancy, early prenatal care with close attention to blood pressure and baby growth are important.  Recommendation**:**   - Starting at 12 weeks, daily low-dose (81mg) aspirin to reduce repeat severe preeclampsia by up to 50%.   **Fetal Maternal Hemorrhage:** Fetal maternal hemorrhage is when the placenta transfers blood from the baby to the mother. In severe cases, the baby can become sick from anemia (low red blood cell count). If the fetus shows signs of distress, you may need to have an urgent delivery. Because fetal maternal hemorrhage is rare, it is unlikely to happen again.  Recommendations:   - There are no specific recommendations to prevent this from occurring. - Weekly non-stress tests are reasonable to monitor the baby beginning at 32 weeks or around the time of the prior event. - A reasonable approach is to pay attention to the baby’s activity and seek early evaluation if the baby is not moving well.   **Uterine Anomaly.** Women with differently shaped uteri, including bicornuate uterus, are at increased risk for preterm birth and for fetal malpresentation (baby being breech).  Recommendation**:**   - Some doctors may recommend weekly injections of 17-hydroxy progesterone and/or monitoring of the length of your cervix if you do have another pregnancy. - It is not clear if these treatments are helpful for women with unusual cases. - Consult with a high-risk pregnancy specialist early in pregnancy if you choose to have another pregnancy.   **Gestational Diabetes.** There is an increased risk of developing diabetes in your next pregnancy if you had gestational diabetes in your previous pregnancy. Two out of three women who have had gestational diabetes during their pregnancy will have it again during a future pregnancy. Between 15% - 70% of women with gestational diabetes will develop diabetes mellitus later in life. Additionally, children of women who had gestational diabetes may be at risk of becoming overweight during childhood, and a higher risk of developing diabetes.  Recommendation**:**   - Have your blood sugar tested for diabetes every 1 – 3 years. Get your blood glucose checked before you get pregnant. - Tell you baby’s doctor that you had gestational diabetes. As your baby grows, his or her blood sugar levels should be checked throughout childhood. - Once you are pregnant again, tell your doctor about your history of gestational diabetes at your first prenatal visit. - Prepare meal plan strategies   **Special Cases.** There are also many other causes of preterm birth including excessive amniotic fluid, fetal abnormalities, poor fetal growth, fetal bleeding and placenta previa. Each has its own repeat rate that ranges from very low to very high.  Recommendation**:** A preconception consult with a Maternal Fetal Medicine specialist is helpful to sort out the medical issues and make individualized recommendations for the next pregnancy.  My Plan: *[Create a Health Plan for Mom for future pregnancies. Use free text to personalize information]*   - Before I decide to become pregnant, schedule a preconception visit with my doctor to help prevent complications - Once you're pregnant, please call your OB/GYN clinic - positive pregnancy test - 1 week late for your period to schedule a pregnancy confirmation 5-1/2 to 6-1/2 weeks after the first day of your last menstrual period - _______________________________________________________________________ - _______________________________________________________________________ - _______________________________________________________________________ - _______________________________________________________________________ |

**Appendix 4. Physician to Physician Letter Template**

[insert date]

Re: ***

Dear Colleague,

Your patient, *** , participated in our pilot program, Moms in the NICU. The purpose of our study is to develop and pilot a NICU-based intervention to engage and empower moms to improve their own health in between and during future pregnancies by creating: a maternal birth story, including accurate information on potential factors associated with preterm birth to help manage future pregnancies, and a health plan for mom to help support actions to improve her own health. We have provided *** with a written record of her birth story and recommended health plan.

*[Insert key events from birth story as told by Mom]*

*[Insert recommendations for future pregnancies]*

These recommendations were made based on discussion with the patient and any records we may have had available at the time, and are not intended to replace a formal perinatal consultation when desired and indicated. If you have any questions or concerns, please feel free to contact us.

Sincerely,

[insert signature]

**Appendix 5. Medical Abstraction Form**

Mom Information:

Name:

DOB:

Gender:

Age:

MRN:

Hx:

Before Delivery/During Pregnancy:

Labor and Delivery:

Baby Information:

Name:

DOB:

Gender:

Age:

MRN:

Comments:

Emotional Distress:

Future Pregnancies:

**Appendix 6. Example of Handout**

| **My Baby’s Birth Story**  A record of what happened during the birth of my baby. |
| --- |
| **Before pregnancy:**   - This was my first pregnancy   **During pregnancy:**   - I received prenatal care with Dr. XXX (Salinas OB) - Had a urinary tract infection at the beginning of my pregnancy - I was diagnosed with gestational diabetes - At 24 weeks, I was seen in an ER in Salinas for spotting (no pain) and was told I was 2 – 3 cm dilated - Was told I had a “thin cervix” - Cerclage was discussed, and I was sent to LPCH for evaluation and treatment - NO cerclage was placed because I was >24 weeks pregnant. Stayed at LPCH for 2 weeks until delivery   **Delivery:**   - Machine showed contractions were occurring, but I didn’t feel them - I received two courses of steroids, but only one dose with last course - Had a vaginal delivery with AROM at LPCH   **My baby’s birth information:**   - Name: XXX - Date of birth: MM/DD/YYYY - Gender: Male - Gestational age at birth: 26 weeks - Birthweight: ____kg - Length: ____ cm - Head circumference: _____ cm - Discharged home from NICU on _____ |
| **Letting My Body Recover**  What my body needs to recover from my recent pregnancy and labor. |
| The length of time your body has to recover between pregnancies is important for all women. The longer your interpregnancy interval, the better chance you have to optimize your body’s nutritional status and ensure healing by allowing your muscles to recover. Mothers who delivery prematurely and have another birth with 18 months have a much higher risk of a repeat preterm birth.  *Recommendation****:*** Family planning using highly effective contraceptives for the next year is strongly recommended. These methods include intrauterine devices (IUDs) and birth control implants. Birth Control pills can also be considered.  *My Plan****:*** *I am currently using Depo Provera shot every 3 months. I would like to have an implant inserted in my arm.*   - *I will call my doctor and set up an appointment to insert a birth control implant in the office.* - *I plan to wait at least one year before trying to conceive again.* |

| **Taking Care Of My Health Now**  What do I need to do to take care of my health right now? |
| --- |
| ***Diabetes***. Gestational diabetes is high blood sugar (glucose) that is first diagnosed during pregnancy. Having gestational diabetes in your last pregnancy increases your risk of having gestational diabetes in another pregnancy and of having type 2 diabetes later in life. While you may not have any symptoms of high blood sugar, diabetes can lead to many health problems, such as high blood pressure, heart disease and kidney disease.  *Recommendations*: Because you had gestational diabetes while you were pregnant, you should be retested for diabetes after your baby is born. Please have the test approximately 6 weeks after your baby’s birth date or _________, and every 1-3 years after giving birth. Breastfeeding is helpful for reducing your risk of developing type 2 diabetes after pregnancy. Eating healthy foods and being active every day can also help to get to and stay at a healthy weight to help reduce your risk of getting type 2 diabetes.  *My Plan*:   - *Call my doctor’s office to schedule a screening test (fasting plasma glucose or oral glucose tolerance test) for diabetes about 6 weeks after my baby’s birth. Remember not to eat anything after midnight of the night before my scheduled test.* - *Remember to get screened every 1-3 years. More frequent screening is needed if I have prediabetes.* |
|  |
| ***Cervical Insufficiency***. In some women, the cervix can shorten and open too early in pregnancy with little or no labor pains. Early additional screening for signs of cervical insufficiency during the early part of your next pregnancy may help to identify potential interventions to reduce recurrent preterm birth.   - Good news! Most likely won’t happen again - Per MFM, for most women it doesn’t happen with a second pregnancy - To reduce the chances of this happening again: - Don’t get pregnant again too soon - Think about contraception: using Depo-Provera right now, thinking about switching to Nexplanon - Recommend approx. 12 months before start trying again   *Recommendation****:***   - Starting at 16 weeks, check cervix with US biweekly till 24 weeks to get measurement - If cervical length < 25 mm, put in stitch - Begin 17 OH progesterone 250mg IM weekly till 36 weeks - Alternative: Cerclage at 13 weeks - at least half the women don’t need it - more aggressive approach |
|  |
|  |

| **A Plan For My Health**  What I can do now to act on these recommendations |
| --- |
| To reduce the chances of this happening again:   - Don’t get pregnant again too soon - Think about contraception: using Depo-Provera right now, thinking about switching to Nexplanon - Recommend approx. 12 months before start trying again |

**Appendix 7. Co-designed Health Plan Template**

After reviewing my plan for letting my body recover, taking care of my health now, and planning for future pregnancies, here are the actions I plan to take now:

Realistically, I can get

this done by:

_______________________

**Appendix 8. Database**

| Private Health Information for Moms | | | | |  | | |  |
| --- | --- | --- | --- | --- | --- | --- | --- | --- |
|  |  | |  | |  | | |  |
|  |  | |  | |  | | |  |
| Items | Participant | | Participant | | Participant | | |  |
| **Project ID** | 1 | | 2 | |  | | |  |
| **Mother's Name** | Malia Doe | | Jennifer Roe | |  | | |  |
| **Mother's DoB** | 10/12/86 | | 1/1/97 | |  | | |  |
| **Baby's Name** | Sasha | | Jo | |  | | |  |
| **Baby's DoB** | 3/25/19 | | 2/28/19 | |  | | |  |
| **Contact Information** | phone 650-867-5309 | | email jroe@jroe.com | |  | | |  |
| **Follow Up Date** | 5/15/19 | | 4/25/19 | |  | | |  |
| **Medical Record Number (optional)** | 314432827356564 | |  | |  | | |  |
|  |  | |  | |  | | |  |
| Project ID | | 1 | | 2 | |  |  | |
| Mother Race/Ethnicity | | Asian | | White | |  |  | |
| IPI for most recent pregnancy (what units?) | | 6 months | | 8 months | |  |  | |
| Baby's gestational age at birth (weeks) | | 30 | | 28 | |  |  | |
| Medical expert meeting date | | 3/30/19 | | 3/31/19 | |  |  | |
| Baby's GA on meeting date (weeks) | | 34 | | 32 | |  |  | |
| Contraception prior to maternal discharge? (y/n) | | n | | y | |  |  | |
| If no, contraception received by now? (y/n) | | y | |  | |  |  | |
| Successful six week follow up (y/n) | | y | | y | |  |  | |
| Made postpartum OB follow up appt (y/n) | | y | | y | |  |  | |
| Barriers to making OB follow up? (y/n) | | y | | n | |  |  | |
| Kept postpartum OB follow up appt (y/n) | | n | | n | |  |  | |
| Barriers to keeping OB follow up? (y/n) | | y | | y | |  |  | |
| Made health issue appts? (y/n) | | y | |  | |  |  | |
| Number of health issue appts planned | | 3 | | 0 | |  |  | |
| Number of health issue appts made | | 2 | | 0 | |  |  | |
| Barriers to making health issue appts? (y/n) | | y | | n | |  |  | |
| Kept health issue appts? (y/n) | | y | |  | |  |  | |
| Number of health issue appts kept | | 2 | |  | |  |  | |
| Barriers to keeping health issue appts? (y/n) | | y | | n | |  |  | |
